# Changes in Physicochemical Properties, Volatile Profiles, and Antioxidant Activities of Black Apple During High-Temperature Fermentation Processing

**DOI:** 10.3389/fnut.2021.794231

**Published:** 2022-02-08

**Authors:** Zuoyi Zhu, Yu Zhang, Wei Wang, Suling Sun, Junhong Wang, Xue Li, Fen Dai, Yunzhu Jiang

**Affiliations:** Institute of Agro-Product Safety and Nutrition, Zhejiang Academy of Agricultural Science, Hangzhou, China

**Keywords:** apple, high-temperature fermentation processing, physicochemical properties, volatile profiles, antioxidant activity

## Abstract

Black apple is a new elaborated product obtained from whole fresh apple through fermentation at controlled high temperature (60~90°C) and humidity (relative humidity of 50~90%). The appearance, color, texture, and taste of black apple changed dramatically compared with those of fresh apple. In this study, changes in the physicochemical and phytochemical properties, volatile profiles, and antioxidant capacity of apple during the fermentation process were investigated. Results showed that the browning intensity and color difference increased continuously during the whole 65-day fermentation process (*p* < 0.05). Sugars decreased in the whole fermentation process (*p* < 0.05), whereas the contents of organic acids increased first and then decreased with prolonged 35 days of fermentation (*p* < 0.05). Total polyphenol content of black apple showed an increase of 1.5-fold as that of fresh apple, whereas 12 common polyphenolic compounds present in fresh apple decreased dramatically in the whole fermentation process (*p* < 0.05). The analysis of flavor volatiles showed that high-temperature fermentation decreased the levels of alcohols and esters and resulted in the formation of furanic and pyranic compounds, which are the main products of Maillard reaction (MR). Antioxidant activities of black apple were enhanced compared with those of fresh apple, and results indicated that the enhancement of antioxidant activities was related to the polyphenols and products of MR.

## Introduction

Nowadays, apple is one of the most consumed fruits worldwide. The popularity of apple all over the world is not only due to its pleasant flavor and nutritional value, but also a due to a significant source of dietary bioactive compounds (1). The dietary intake of apple has been shown to have significant benefits to human health, including lowering the risk of cancer, cardiovascular diseases, obesity, type-2 diabetes, and inflammatory disorders (2–5). Recently, China has become the largest apple producer among the world, with apple planting area and yield accounting for approximately 50% of the world. The production of apple in China was 42.43 million tons in 2019. However, the main consumption way of apple is fresh fruit. Dried apple, apple juice, and cider make up only a small proportion of the apple consumption (6, 7). With the expansion of apple planting scale, market saturation, and lack of deep processing technology, apple supply in excess of demand happens all the time, leading to serious economic losses of fruit farmers. In addition, in the process of apple cultivation, due to the planting technology, management, climate, and environment etc., a part of the apples have defects in shape, color, and appearance. Such defects greatly affect the sale of fresh apples. Therefore, it is of great significance to develop advanced processing technologies for apple.

Thermal processes are commonly used in food manufacturing to improve the sensory quality of foods, such as colors, textures, and tastes, as well as to improve the storage performance of foods. This will cause chemical changes to form new compounds that are not originally present in foods. Black garlic is a thermal-processed product, which is prepared through natural fermentation of fresh garlic cloves at high temperature and humidity for a long time (8). Black garlic has been a popular food particularly in China, Korea, and Japan (9). After high-temperature fermentation processing (HTFP), the garlic cloves change from white to black with the texture becoming soft and elastic. The taste of black garlic is sweeter without the pungent odor (10). Many studies have shown that black garlic exhibits a variety of biological activities, such as antioxidant, hepatoprotective, neuroprotective, antiallergic, antidiabetic, and anticancer activities (11–15). Although the application of HTFP on garlic has been intensively studied and widely used, HTFP on other plant foods such as onion, potato, apple, and peach has not been tried yet. In view of the high-quality health benefits of black garlic, HTFP can be a potential alternative to traditional thermal processing technologies to develop safe, tasty, nutritive, and functional food products.

In our previous study, the HTFP technology of black garlic was carried out on apple as reference (16). A new fermented product with characteristic black appearance, named as black apple, was obtained. The texture of the final products becomes soft and sticky, with a sweet-sour flavor. At the same time, many nonenzymatic reactions such as Maillard reaction (MR) and caramelization reaction, occurred during the HTFP process of apple. These reactions will definitely cause changes in the physicochemical properties and biological activities of apple. To the best of our knowledge, there is no information available about the changes of apple during the HTFP process. However, the influences of thermal process on the physicochemical properties, bioactive compounds, volatile profiles, and biological activities of apple are still unknown. Therefore, the objective of this study was to investigate the changes in the physicochemical and phytochemical properties of apple, including color, browning intensity, pH, the contents of moisture, sugars, organic acids, phenolic and volatile compounds, during the 65-day HTFP process. The antioxidant activities of apple during the HTFP process were also studied by 2,2′-azino-bis(3-ethylbenzothiazoline-6-sulfonic acid) diammonium salt (ABTS), 2,2′-diphenyl-1-picrylhydrazyl (DPPH), hydroxyl radicals, FRAP, and Fe^2+^-chelating assays. Furthermore, the correlation between physicochemical properties and antioxidant activities of processed apple was also evaluated.

## Materials and Methods

### Chemicals

2,2′-Azino-bis(3-ethylbenzothiazoline-6-sulfonic acid) diammonium salt, DPPH, 2,3,5-triphenyltetrazolium chloride (TPTZ), 6-hydroxy-2,5,7,8-tetramethylchromane-2-carboxylic acid (Trolox), 3-nonanone as internal standard (IS), and ferrozine were purchased from Sigma Chemical Co. (St. Louis, MO, United States). Sorbitol, glucose, fructose, sucrose, formic acid, acetic acid, lactic acid, malic acid, citric acid, gallic acid (GA), protocatechualdehyde (PRO), (+)-catechin (CAT), chlorogenic acid (CHL), caffeic acid (CAF), syringic acid (SYR), epicatechin (EPI), *p*-coumaric acid (*p*-CUM), rutin (RUT), phlorizin (PRI), quercetin (QUE), and phloretin (PRE) were purchased from Shanghai Yuanye Biotechnology Co., Ltd. (Shanghai, China). All other reagents used were of analytical grade.

### Samples

Apple samples were kindly provided by Zhejiang Shengyuan Agricultural Science & Technology Co., Ltd. The whole fresh Fuji apples were cleaned and fermented with activated lactic acid bacteria for 24 h and then manufactured in a fermentation bin at controlled temperature (60~90°C) and humidity (relative humidity of 50~90%) for 65 days. The fresh apples were used as the reference blank. Samples were removed after 5, 15, 30, 45, and 65 days of high-temperature fermentation treatment. Moisture content, color, and pH values were determined immediately after sample removing. Then, each sample was divided into two subsamples. One was lyophilized using a freeze dryer and then ground into powder by a high-speed grinding powder machine. The other was frozen with liquid nitrogen and stored at −80°C until volatile profile analysis.

### Determination of Color

The apples were sliced and the inside color was measured using a portable CR-410T colorimeter (Konica Minolta, Japan). The total color difference (Δ*E*) was calculated by the following formula (17):


$$\Delta E\;=\;{\left(\Delta L\right)}^{2}\;+\;{\left(\Delta a\right)}^{2}\;+\;{\left(\Delta b\right)}^{2}$$

where Δ*L*, Δ*a*, and Δ*b* represent the difference between processed apple and fresh apple sample (0 day) on *L, a*, and *b* values, respectively.

### Moisture Contents Measurement

Moisture contents were measured according to the vacuum drying method described in Chinese standard GB5009.3-2016 (18) with a DZF-6050 vacuum drying oven (Shanghai Yiheng Scientific Instrument Co., Ltd., China).

### Determination of pH Values

Five grams of the homogenized sample was added to 20 mL distilled water and vortexed at a speed of 2,000 rpm for 3 min. Then, the homogeneous samples were filtered before the pH measurement. A digital pH meter (PHS−3C, Shanghai Precision Instrument Co., Ltd, China) was used to measure the pH values of samples.

### Sample Pretreatment

One gram of lyophilized sample was extracted with 100 mL distilled water by ultrasonic extraction at 25°C for 60 min. The mixture was then centrifuged at 4,000 g for 10 min. The liquid supernatant was collected and diluted for further browning intensity, sugars, and organic acids determination and antioxidant analysis.

### Determination of Browning Intensity

The browning intensity (K420) of apple samples was determined by the method described by Lertittikul et al. (19). The absorption values of apple samples at 420 nm were measured against a blank (distilled water) by a N5000 spectrophotometer (Shanghai Youke Instrument Co., Ltd, China).

### Determination of Sorbitol, Glucose, Fructose, and Sucrose

The contents of sugars were determined by ion chromatography with pulsed amperometric detection (IC-PAD). The determination was performed on a Thermo Fisher Scientific (Sunnyvale, CA, United States) ICS-3000 ion chromatograph. CarboPac PA10 guard column (50 mm × 4 mm i.d., 10 μm) and analytical column (250 mm × 4 mm i.d., 10 μm) were used for separation. The elution mode was as follows: 15 mM NaOH from 0 to 12 min; 100 mM NaOH from 12.1 to 17 min; and 15 mM NaOH from 17.1 to 22 min. The flow rate was 1.0 mL/min. Column temperature was set at 30°C. The conditions of PAD were the same as the published method (20). The results were expressed as g/100 g of dry matter (DM).

### Determination of Organic Acids

The contents of organic acids were determined by IC with conductivity detection. The determination was also performed on a Thermo Fisher Scientific ICS-3000 ion chromatograph. Guard column (IonPac AG11-HC, 50 mm × 4 mm i.d., 9 μm) and analytical column (IonPac AS11-HC, 250 mm × 4 mm i.d., 9 μm) were used for separation. Gradient elution mode was employed as follows: 1 mM NaOH from 0 to 7 min; 1–30 mM NaOH from 7.1 to 25 min; keeping at 30 mM NaOH from 25.1 to 30 min, and 1 mM NaOH from 30.1 to 35 min. Suppression current was set at 75 mA. The flow rate was 1.0 mL/min, and the column temperature was set at 30°C. The results were expressed as g/kg of DM.

### Total Polyphenol Content (TPC) and Polyphenolic Compounds

The Folin-Ciocalteu method was used to measure the TPC of apple samples. Briefly, 1 g of lyophilized sample was ultrasonically extracted with 10 mL of acetic acid–water–methanol (1:29:70, v/v/v) for 60 min. The mixture was then centrifuged at 4,000 g for 5 min and the liquid supernatant was collected. A re-extraction of the residue was performed. The supernatants were combined and made up to 20 mL with water. The apple extract (1 mL) was mixed with 5 mL of Folin-Ciocalteu reagent. After 5 min, 4 mL of 7.5% Na_2_CO_3_ was added. The mixture was left to react for 60 min in the dark. The absorbance was measured at 765 nm. Gallic acid (10–100 mg/L) was used as standard for the quantification of TPC. The content of TPC was expressed as mg GA equivalents per g of DM (mg GAE/g).

High-performance liquid chromatography-electrochemical detection (HPLC-ECD) was used to determine the polyphenols in apple samples followed by a prior study carried out in our laboratory (21), with minor modifications. Four milliliters of the extraction solution above was evaporated using a stream of nitrogen to 1 mL before injection. The analytical column was an Agilent XDB C18 column (250 mm x 4.6 mm i.d., 5 μm) maintained at 35°C. The mobile phase consisted of phosphoric acid (0.05%, v/v) as solvent A and methanol as solvent B. The gradient program was as follows: 0 min (95% A + 5% B); 20 min (80% A + 20% B); 40 min (70% A + 30% B); 50 min (55% A + 45% B) and kept for 15 min; 65–70 min (95% A + 5% B). The flow rate was 1.0 mL/min. ECD was performed on a glassy carbon electrode and was set at 1.0 V in oxidative mode. The results were expressed as μg/g of DM.

### Determination of the Volatile Profile

Headspace solid-phase microextraction followed by gas chromatography-tandem mass spectrometry (HS-SPME/GC-MS/MS) was used to determine the volatile compounds. Frozen samples were melted and homogenized. Weighed samples (equivalents of 0.5 g DM) were transferred to a 20-mL headspace vial. Distilled water was added to 10 mL, and then 2 g of NaCl and 5 μL of internal standard (IS, 3-nonanone/methanol at 1:50,000, v:v) were added. The solution was heated for 20 min at 40°C with constant stirring, and then a 65-μm DVB/CAR/PDMS fiber (Supelco, PA, United States) was exposed to the headspace of the sample for 20 min at 50°C for SPME extraction.

The Agilent 8890 GC system was interfaced with an Agilent 7000D triple quadrupole mass spectrometer (Agilent, CA, United States). A HP-5 (30 m × 0.25 mm i.d. × 0.25 μm)-fused silica capillary column (Agilent, CA, United States) was used for separation. After HS-SPME extraction, the fiber was introduced into the GC injector port for the thermal desorption of analytes at 250°C for 6 min. A split injection was used with the split ratio of 2:1. The following chromatographic program was used: 40°C kept for 5 min, increased 2°C/min−60°C and kept for 2 min, increased 3°C/min−120°C, increased 5°C/min−150°C, and then increased 8°C/min−250°C with 5-min hold, with a total run time of 60 min. The flow rate was kept at 1 mL/min with helium as carrier gas. The quadrupole mass detector, ion source, and transfer line temperatures were set at 150, 230, and 250°C, respectively. MS acquisition was carried out in full scan mode (in the range m/z 50–500 amu) with electronic impact (EI) mode at 70 eV. The identification of volatile compounds was performed by matching the mass spectra with the NIST17 data library with a similarity higher than 80% and by comparing the Kovats index (KI) values (22) with the values reported in the NIST library. The relative content (RC) of each volatile compound was calculated by the ratio between the peak area of volatile compound and IS.

### Antioxidant Activity

#### ABTS Radicals Assay

The ABTS radical scavenging activity was evaluated as follows (23). Briefly, ABTS radicals were produced by reacting 7 mM ABTS stock solution with 2.45 mM potassium persulfate. The mixture was left in the dark for 12–16 h before use. The ABTS solution was diluted with 5 mM phosphate-buffered saline (pH 7.4) to an absorbance of 0.70 ± 0.02 at 730 nm. Twenty microliters of sample solution was added to 280 μL of the diluted ABTS solution on a transparent 96-well polystyrene microplate. The absorbance was measured after 20 min using an AMR-100 microplate reader (Hangzhou Allsheng Instrument Co., Ltd, China). ABTS radical scavenging activity was calculated as follows: scavenging rate (%) = [1– (A_1_-A_2_)/A_0_] × 100, where A_0_ was the absorbance of ABTS solution, A_1_ was the absorbance in the presence of sample and ABTS radicals, and A_2_ was the background absorbance of sample.

#### The DPPH Radical Assay

The DPPH radical scavenging activity was measured according to the method reported by Braca et al. (24) with a slight modification. Briefly, 0.1 mL of sample solution was mixed with 2.9 mL of 0.2 mM DPPH–ethanol solution. The mixture was left in the dark for 10 min. Absorbance was measured at 517 nm using N5000 spectrophotometer. DPPH radical scavenging activity was calculated as follows: Scavenging rate (%) = [1– (A_1_-A_2_)/A_0_] × 100, where A_0_ is the absorbance of DPPH solution, A_1_ is the absorbance in the presence of sample and DPPH radicals, and A_2_ is the background absorbance of sample.

#### Hydroxyl Radical Assay

The hydroxyl radical scavenging activity was determined according to the method published by Chen et al. (25). The reaction mixture consisted of 0.1 mL of H_2_O_2_ (20 mM), 0.1 mL of FeSO_4_ (10 mM), 0.1 mL of salicylic acid–ethanol solution (10 mM), 2.5 mL deionized water, and 0.2 mL of sample solution. The mixture was incubated at 37°C for 1 h, and the absorbance was measured at 510 nm. The hydroxyl radical scavenging activity was calculated as follows: scavenging rate (%) = [1– (A_1_-A_2_)/A_0_] × 100, where A_0_ is the absorbance of hydroxyl radicals, A_1_ is the absorbance in the presence of sample and hydroxyl radicals, and A_2_ is the background absorbance of sample.

#### The FRAP Assay

The FRAP ability was estimated according to the procedure described in the study by Benzie and Strain (26) with a slight modification. The FRAP reagent consisted of 2.5 mL of 10 mM TPTZ solution in 40 mM HCl, 2.5 mL of 20 mM FeCl_3_.6H_2_O, and 25 mL of 0.3 M acetate buffer at pH 3.6. In brief, 280 μL of FRAP reagent was mixed with 20 μL of sample. The mixture was left in the dark for 10 min. The absorbance of the mixture was measured at 595 nm using AMR-100 microplate reader. Trolox solutions (0.1–1.0 mM) were used to perform the calibration curve. Results were expressed as μM equivalents of Trolox per g sample (DM).

#### Measurement of Fe^2+^-Chelating Ability

The Fe^2+^-chelating ability was determined according to the method published by Decker and Welch (27). In brief, 0.5 mL of the sample solution was added to 0.1 mL of 0.2 mM FeCl_2_ and 0.2 mL of 0.5 mM ferrozine solutions. After reaction for 10 min, the absorbance at 562 nm was measured. The Fe^2+^-chelating ability was calculated as follows: Chelating rate (%) = [1– (A_1_-A_2_)/A_0_] × 100, where A_0_ is the absorbance of the blank control group, A_1_ is the absorbance of the test group, and A_2_ is the background absorbance of the sample.

### Statistical Analysis

All experiments in this study were performed three times. Experimental data were reported as mean values with SD. A *p* < 0.05 was used to indicate significant differences, and a *p* < 0.01 was used to indicate extremely significant differences. The statistical program SPSS 19.0 (Armonk, NY, United States) was used to analyze the data.

## Results and Discussion

### Changes in Appearance, Color, Moisture Content, pH, and Browning Intensity

**Figure 1:**
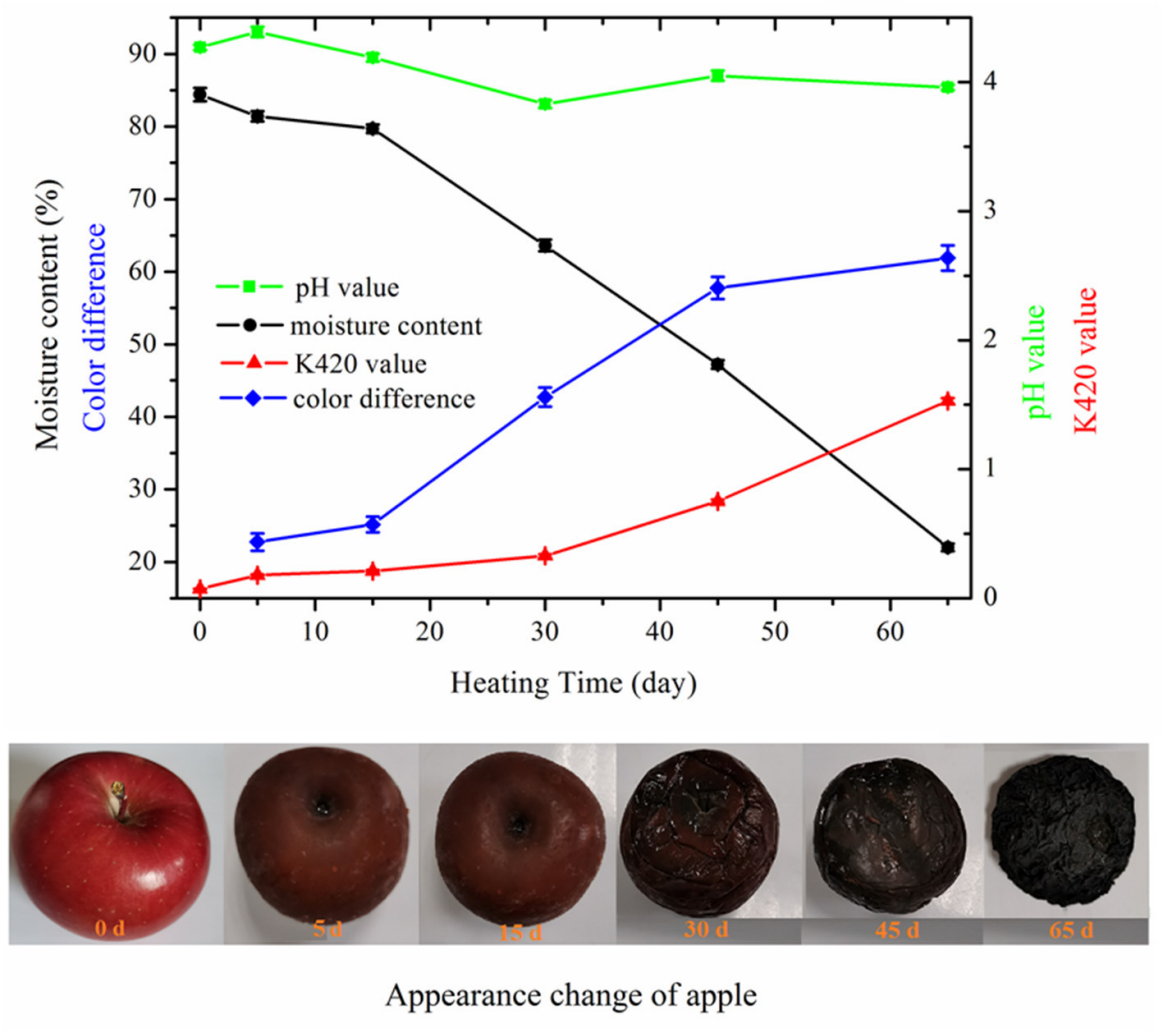
Changes in appearance, moisture content, color difference, browning intensity, and pH of apple during 65-day HTFP process.

When fresh apple underwent high-temperature fermentation, the color of apple turned brown first and then changed to dark brown and black gradually (Figure 1). This change was mainly due to the nonenzymatic browning reactions and the formation of high molecular weight (MW) compounds, such as melanoidins, resulting from MR. The apples became soft at day 15 of fermentation since the texture began to change. After that, wrinkles appeared on the surface of apple due to the moisture loss. The texture of black apple finally became sticky and chewy. The total color difference (ΔE) increased from 22.74 ± 1.21 to 61.87 ± 1.74 over fermentation time of 5–65 days. The moisture content of apple decreased slowly in the first 15 days due to the high relative humidity of HTFP and then decreased dramatically from 79.7 ± 0.6% to 22.0 ± 0.5% of the final products. The pH value did not vary much throughout the whole fermentation process. The pH value of raw apple was 4.27 and decreased eventually to 3.96 of the final products. The pH decrease of black apple was not as apparent as that of black garlic. According to the reported study by Zhang et al. (9), the pH value decreased from 6.25 to 4.25 with whole heating process. The decrease of pH was probably due to the organic acids developed by the fermentation and produced a sour taste mouthfeel of black apple. The browning intensity (K420) of apple extract showed a sustained increase with the increasing HTFP time. In the initial stage of 30 days, it increased slowly from 0.07 ± 0.003 to 0.33 ± 0.01, whereas it increased sharply from 0.33 ± 0.01 to 1.53 ± 0.02 in the latter stage. As reported, the K420 is an important index of MR and is known to correlate with the antioxidant activity of MR systems (28). These results indicated that vigorous MR occurred in the latter stage of HTFP, leading to the formation of high MW compounds like melanoidins.

### Changes in Sugars

**Figure 2:**
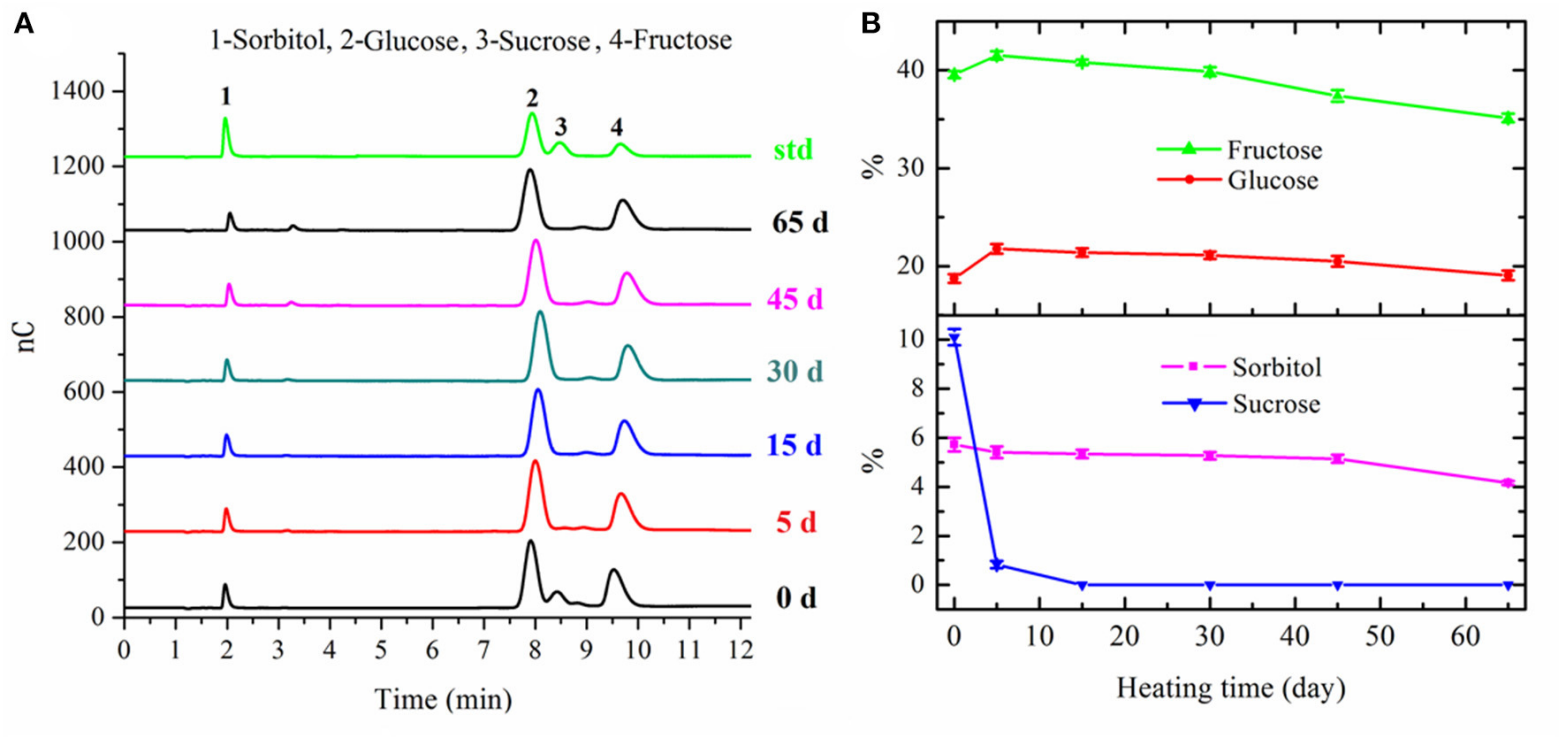
Chromatograms of sugars in apple samples with different fermentation days **(A)** and changes in sugars of apple during 65-day HTFP process **(B)**.

The sugars in fresh apple mainly consisted of sorbitol (5.72 ± 0.28 g/100 g DM), fructose (39.55 ± 0.34 g/100 g DM), glucose (18.75 ± 0.43 g/100 g DM), and sucrose (10.10 ± 0.33 g/100 g DM), respectively. With regard to sucrose, the content of this disaccharide decreased extremely to 0.83 ± 0.15 g/100 g DM in apple after 5 days' fermentation, and then degraded into fructose and glucose completely after 15 days' fermentation (Figure 2). The degradation of sucrose and other polysaccharides resulted in an increase for both fructose and glucose after 5 days' fermentation. After that, both the contents of fructose and glucose decreased gradually in the following fermentation process. The content of fructose decreased to 35.12 ± 0.44 g/100 g DM in the final black apple product. However, the content of glucose in the final black apple product increased a little bit compared with that of fresh apple. The consumption of fructose and glucose in apple during the fermentation process could mainly be ascribed to MR and bioconversion of sugars into organic acids. These results also suggested that the reactivity of fructose was higher than that of glucose, presumably because fructose had a higher proportion of open chain form than glucose (29). As shown in Figure 2, a slight decrease in the content of sorbitol was observed during 0–45 days of fermentation. The content of sorbitol decreased from 5.14 ± 0.16 g/100 g DM to 4.16 ± 0.08 g/100 g DM during the last 20 days' fermentation.

### Changes in Organic Acids

**Figure 3:**
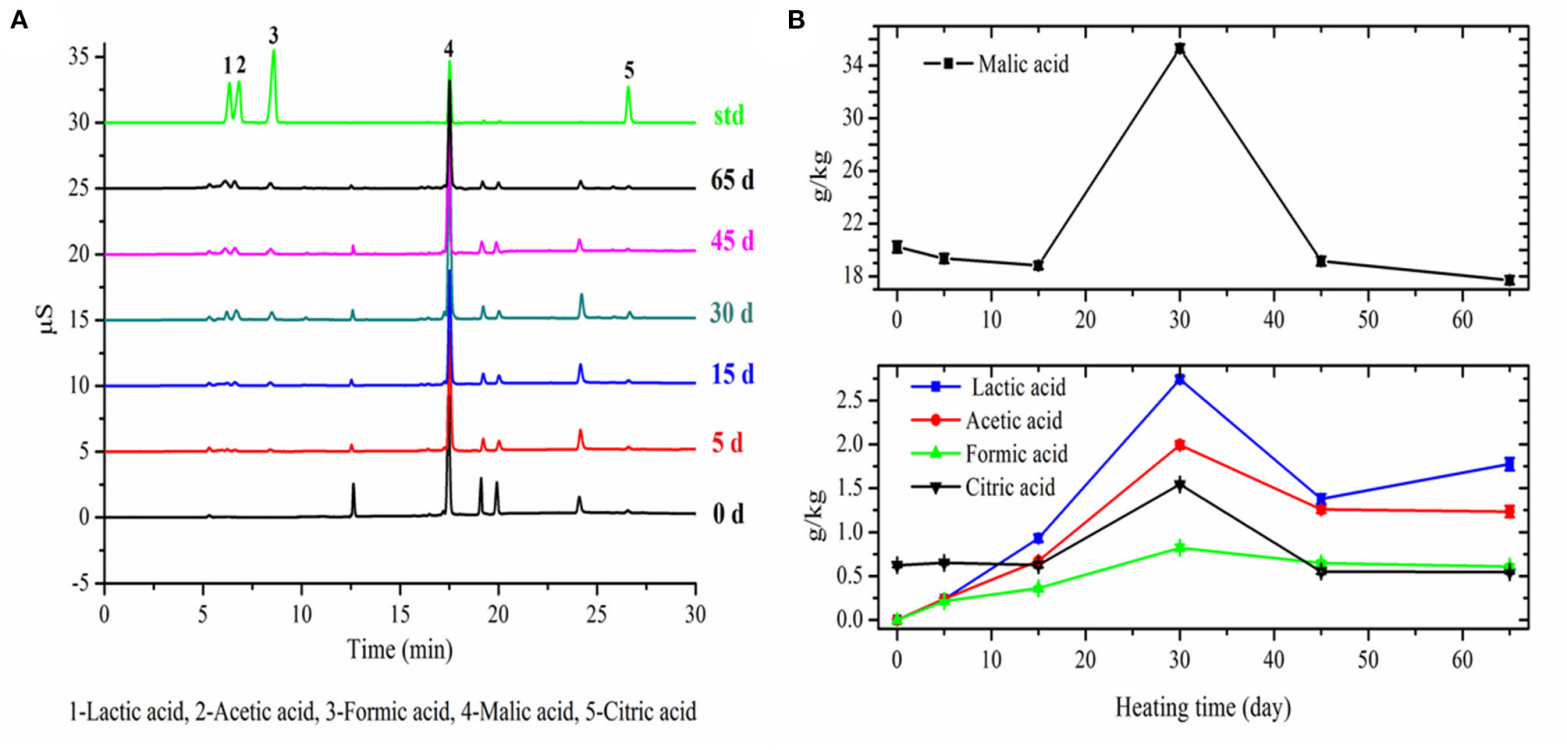
Chromatograms of organic aids in apple samples with different fermentation days **(A)** and changes in organic aids of apple during 65-day HTFP process **(B)**.

The variation trends of five organic acids' contents in apple with fermentation time were similar (Figure 3). Among them, lactic acid, acetic acid, and formic acid were not present in fresh apples. The contents of these three organic acids were remarkably increased to a maximum of 2.74 ± 0.056 g/kg DM, 1.99 ± 0.044 g/kg DM, and 0.82 ± 0.033 g/kg DM in apples within 30 days' fermentation, respectively, and then, there was an obvious decrease at day 45 of fermentation. The content of malic acid ranged from 20.23 ± 0.43 g/kg DM in fresh apple to 17.70 ± 0.31 g/kg DM in the final black apple. The content of malic acid decreased slowly during the first 15 days, remarkably increased to a maximum of 35.30 ± 0.32 g/kg DM at 30 days' fermentation, and then decreased sharply. The content of citric acid in apple was extremely low compared with malic acid, and its variation trend in apple with fermentation time was similar as malic acid. The increase of the organic acids during the first half-stage of fermentation could be attributed to the bioconversion of sugars into organic acids and the decrease during the latter half stage of fermentation could be attributed to the volatilization and degradation of organic acids under high temperature and lower relative humidity. The results of organic acids were in agreement with the pH results.

### Changes in TPC and Polyphenolic Profiles

**Figure 4:**
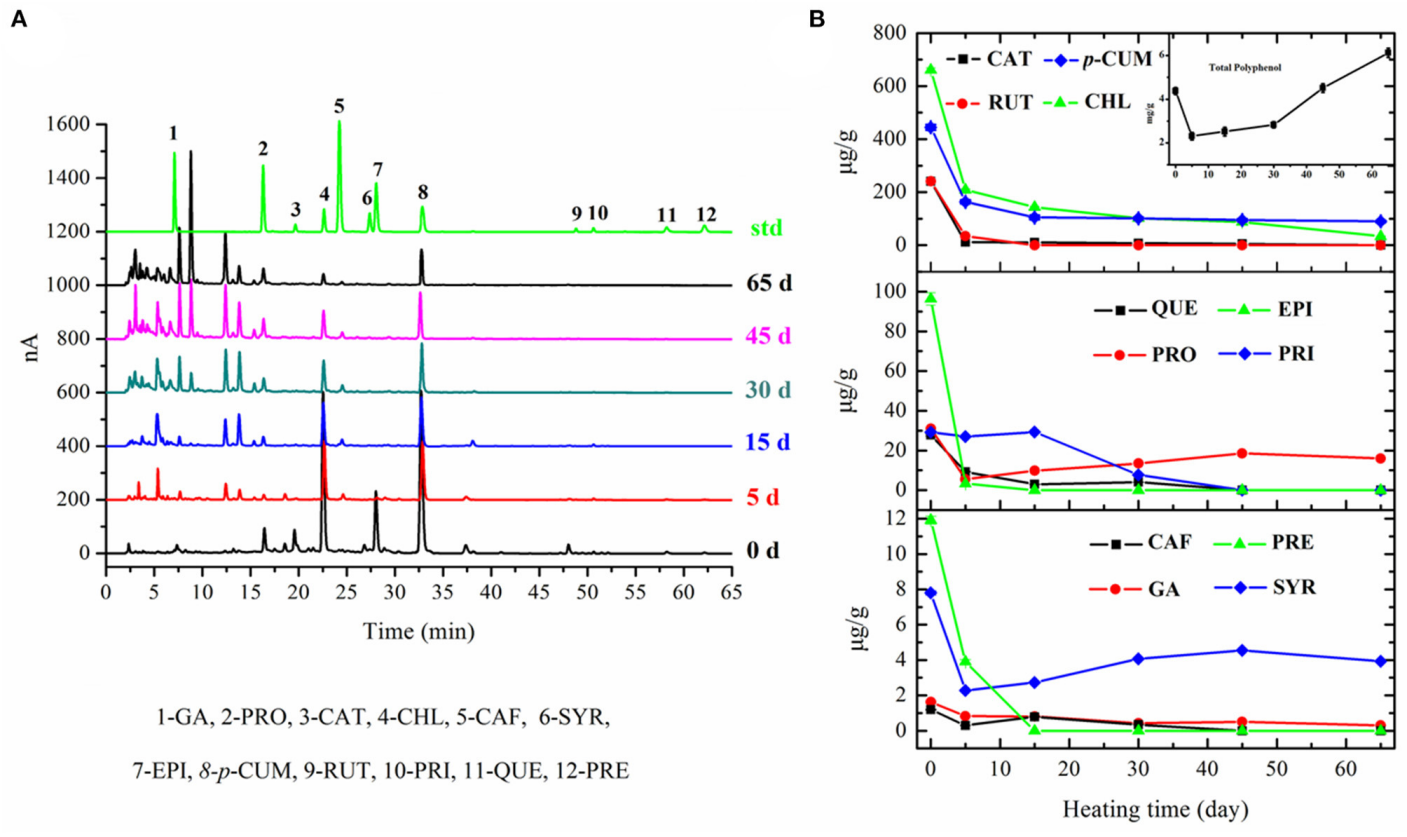
Chromatograms of polyphenols in apple samples with different fermentation days **(A)** and changes in TPC (inset) and polyphenolic profiles of apple during 65-day HTFP process **(B)**.

The results showed that the TPC of fresh apple was 4.27 ± 0.16 mg GAE/g DM. The TPC of apple decreased to 2.31 ± 0.17 mg GAE/g DM after the initial 5 days' fermentation, increased slowly in the following 25 days, and then increased sharply during the latter half-stage of fermentation (inset of Figure 4). The TPC in black apple was 6.22 ± 0.21 mg GAE/g DM and showed an increase of 1.5-fold as that in fresh apple. Previous black garlic studies have described an increase in polyphenol content of about 3-fold, compared to raw garlic (30).

The transformation process of fresh apple into black apple also caused changes in the phenolic acid and flavonoid profiles. According to the results in Figure 4, the main polyphenols present in fresh apple are CHL (660.98 ± 18.29 μg/g DM), *p*-CUM (440.86 ± 10.23 μg/g DM), RUT (240.90 ± 10.66 μg/g DM), CAT (240.47 ± 5.87 μg/g DM), and EPI (96.36 ± 6.11 μg/g DM). There was a great loss for almost all 12 polyphenols after 5 days' fermentation when compared with fresh apple, except PRI. The levels of most compounds were continuously decreased in apples as the increasing fermentation time to day 30, and then kept nearly at a constant in the following fermentation time to the final products, except PRO and SYR. The amount of PRI was basically unchanged in the initial 15 days of fermentation and then decreased to zero at day 45. Only *p*-CUM, CHL, PRO, and SYR existed in the final black apple products with contents of 89.60 ± 4.21 μg/g DM, 33.63 ± 2.14 μg/g DM, 15.99 ± 0.39 μg/g DM, and 3.93 ± 0.23 μg/g DM, respectively. Regarding the decrease in phenolic acids, the results of some studies about black garlic are in accordance with ours (31). Studies of citrus peel extract also showed that the thermal process decreased the total phenolic acids content (32). The decreased concentration of phenolic acids in black apple can be due to the fact that some phenolic acids are unstable when they undergo a thermal process. However, the results of flavonoids in this study were different from those reported by Martinez-Casas et al. (31). The contents of flavonoids decreased during the HTFP process in our study. As reported, an increase of EPI was observed in the black garlic and no difference was found in the amount of apigenin between black garlic and raw garlic. In fact, some flavonoids, like RUT, break down easily in the light and high temperature. The evolution of phenolic compounds in foods during the thermal process is ambiguous, with great differences depending on the products. The contents of 12 analyzed polyphenolic compounds originally found in fresh apples decreased dramatically in the black apple, whereas the TPC of black apple increased compared with that of fresh apple. This may be due to the fact that large amounts of new polyphenolic compounds were produced. According to the HPLC chromatograms of the processed apple, a lot of unidentified peaks appeared, which remain to be identified by HPLC-MS in our following research study.

### Volatile Profiles

Forty-eight volatile compounds were identified in apples and were classified into nine groups: alcohols, esters, acids, and carbonyl, terpenoid, furanic, pyranic, pyrrole, pyridine compounds (Table 1). In fresh apple, 30 volatile compounds consisting of eight alcohols (42.02%), 14 esters (26.78%), four carbonyl compounds (11.20%), and four terpenoid compounds (20.00%) were detected. These results were in good agreement with those reported by Aprea et al. (33). Processed apple presented significant differences in the volatile profiles regarding fresh apple. Only three alcohols and five esters remained at day 5 of fermentation, and most of them showed continuous decreasing as fermentation progressed, except phenylcarbinol, 2-phenylethanol, and 3-methylcyclopentyl acetate. The contents of these three compounds in apple increased first at day 5 of fermentation and then decreased. The temporary increase could be due to the fact that high temperature caused the disruption of the cell wall, resulting in a release of them in the apple. Only 2-ethyl-1-hexanol and 3-methylcyclopentyl acetate were present in the final black apple products. For the four carbonyl compounds found in fresh apple, only hexanal was still detected in apple at day 5 of fermentation and its amount continuously decreased. Besides, β-damascenone was first found at day 5 of fermentation in apple and its content fluctuated in the following fermentation time. 4-cyclopentene-1,3-dione was detected at day 30 of fermentation of apple and its contents showed an increase in the following fermentation time. Four terpenoid compounds, namely, D-limonene, α-cedrene, α-farnesene, and cedrol, were found in fresh apple. The content of D-limonene increased in the initial 15 days of fermentation and then decreased in the latter stage of fermentation. The contents of the other three compounds decreased during the whole fermentation process. Another terpenoid compound, γ-terpinene, was first detected at day 5 of fermentation of apple, whereas it was not found in the final black apple as cedrol. For acids, only 2-methyl-butanoic acid was detected in apple from the fifth day of fermentation with its content decreasing during the fermentation process. Nine furanic compounds, as the main products of MR, were detected in the fermentation process of apple. Among them, seven furanic compounds were first detected at day 5 of fermentation in apple and their concentrations increased as fermentation progressed. Except 2-acetyl-5-methylfuran, the contents of the other six furanic compounds increased sharply as fermentation progressed. The contents of 5-hydroxymethylfurfural, 5-methyl-2-furancarboxaldehyde, 3-furaldehyde, 1-(2-furanyl)-ethanone, 1-(2-furanyl)-2-hydroxy-ethanone, and 2,5-diformylfuran in the final black apple products were 148.5-fold, 108.1-fold, 19.3-fold, 18.8-fold, 18.6-fold, and 14.5-fold compared with those in apples at day 5, respectively. These results are in agreement with those from Molina-Calle et al. (34), who demonstrated that 5-hydroxymethylfurfural increased its concentration with garlic fermentation, confirming the development of MR during garlic fermentation. 2-Methyl-benzofuran and 2-furfuryl-5-methylfuran were only detected in the final products with low amounts. Except furanic compounds, pyranic, pyrrole, and pyridine compounds were also detected in the latter stage of fermentation as the products of MR and only maltol decreased with increasing fermentation time. These new generated volatiles might contribute to the flavor and aroma of baking in black apple.

**Table 1:** Volatile compounds identified in apples with different fermentation time and their relative contents.

**Chemical families**	**Compounds**	**Formula**	${\text{KI}}_{\text{CAL}}^{\text{a}}$	${\text{KI}}_{\text{NIST}}^{\text{b}}$	**RC_**0d**_**	**RC_**5d**_**	**RC_**15d**_**	**RC_**30d**_**	**RC_**45d**_**	**RC_**65d**_**
Alcohols	2-methyl-1-butanol	C_5_H_12_O	734	739	1.764 ± 0.145	–	–	–	–	–
	3-methyl-1-butanol	C_5_H_12_O	735	736	0.172 ± 0.016	–	–	–	–	–
	3-(methylthio)-1-propanol	C_4_H_10_OS	976	981	0.169 ± 0.012	–	–	–	–	–
	2-methyl-6-hepten-1-ol	C_8_H_16_O	995	994	0.131 ± 0.009	–	–	–	–	–
	2-ethyl-1-hexanol	C_8_H_18_O	1,034	1,030	0.648 ± 0.072	0.365 ± 0.029	0.305 ± 0.041	0.250 ± 0.036	0.062 ± 0.005	0.043 ± 0.004
	Phenylcarbinol	C_7_H_8_O	1,036	1,036	0.043 ± 0.005	0.608 ± 0.038	0.342 ± 0.041	–	–	–
	2-phenylethanol	C_8_H_10_O	1,112	1,116	0.084 ± 0.006	0.216 ± 0.026	0.260 ± 0.023	0.102 ± 0.011	–	–
	2-phenoxy-ethanol	C_8_H_10_O_2_	1,219	1,225	0.019 ± 0.003	–	–	–	–	–
Esters	Butyl acetate	C_6_H_12_O_2_	817	812	0.006 ± 0.001	–	–	–	–	–
	2-methylbutyl acetate	C_7_H_14_O_2_	881	880	1.082 ± 0.112	–	–	–	–	–
	3-methylcyclopentyl acetate	C_8_H_14_O_2_	902	905	0.148 ± 0.023	0.748 ± 0.052	0.614 ± 0.056	0.299 ± 0.024	0.216 ± 0.024	0.092 ± 0.011
	Methyl hexanoate	C_7_H_14_O_2_	926	925	0.054 ± 0.006	0.016 ± 0.002	–	–	–	–
	Ethyl hexanoate	C_8_H_16_O_2_	1,003	1,000	0.039 ± 0.005	0.027 ± 0.003	–	–	–	–
	Hexyl acetate	C_8_H_16_O_2_	1,019	1,011	0.360 ± 0.023	–	–	–	–	–
	Methyl octanoate	C_9_H_18_O_2_	1,128	1,126	0.132 ± 0.017	–	–	–	–	–
	Diethyl succinate	C_8_H_14_O_4_	1,186	1,182	0.044 ± 0.006	0.023 ± 0.002	0.044 ± 0.033	0.026 ± 0.003	–	–
	Methyl salicylate	C_8_H_8_O_3_	1,192	1,192	0.006 ± 0.001	0.006 ± 0.001	0.002 ± 0.0003	–	–	–
	Butyl hexanoate	C_10_H_20_O_2_	1,194	1,189	0.113 ± 0.020	–	–	–	–	–
	Methyl nonanoate	C_10_H_20_O_2_	1,227	1,225	0.029 ± 0.004	–	–	–	–	–
	n-hexyl-2-methylbutanoate	C_11_H_22_O_2_	1,240	1,236	0.014 ± 0.002	–	–	–	–	–
	Isopentyl hexanoate	C_11_H_22_O_2_	1,255	1,252	0.013 ± 0.002	–	–	–	–	–
	2-methylbutyl octanoate	C_13_H_26_O_2_	1,453	1,449	0.011 ± 0.001	–	–	–	–	–
Carbonyl compounds	Hexanal	C_6_H_12_O	803	800	0.172 ± 0.021	0.062 ± 0.005	0.045 ± 0.005	0.037 ± 0.005	0.027 ± 0.003	
	4-cyclopentene-1,3-dione	C_5_H_4_O_2_	884	881	–	–	–	0.033 ± 0.004	0.062 ± 0.005	0.141 ± 0.020
	6-methyl-5-hepten-2-one	C_8_H_14_O	988	986	0.294 ± 0.034	–	–	–	–	–
	(E)-2-octenal	C_8_H_14_O	1,061	1,060	0.363 ± 0.027	–	–	–	–	–
	(E)-2-nonenal	C_9_H_16_O	1,162	1,162	0.029 ± 0.004	–	–	–	–	–
	β-damascenone	C_13_H_18_O	1,386	1,386	–	0.013 ± 0.002	0.005 ± 0.001	0.009 ± 0.001	0.005 ± 0.001	0.006 ± 0.001
Terpenoid compounds	D-limonene	C_10_H_16_	1,028	1,018	1.166 ± 0.078	1.605 ± 0.192	2.770 ± 0.223	1.258 ± 0.021	0.081 ± 0.007	0.090 ± 0.008
	γ-terpinene	C_10_H_16_	1,060	1,060	–	0.034 ± 0.004	0.043 ± 0.004	0.022 ± 0.003	–	–
	α-cedrene	C_15_H_24_	1,411	1,411	0.044 ± 0.005	0.035 ± 0.003	0.034 ± 0.003	0.023 ± 0.004	0.016 ± 0.002	0.011 ± 0.002
	α-farnesene	C_15_H_24_	1,511	1,508	0.322 ± 0.036	0.113 ± 0.014	0.081 ± 0.005	0.013 ± 0.001	0.011 ± 0.001	0.004 ± 0.0003
	Cedrol	C_15_H_26_O	1,606	1,598	0.189 ± 0.015	0.181 ± 0.021	0.052 ± 0.006	0.025 ± 0.003	–	–
Acids	2-methyl-butanoic acid	C_5_H_10_O_2_	864	861	–	0.193 ± 0.023	0.156 ± 0.021	0.087 ± 0.009	0.058 ± 0.007	0.056 ± 0.007
Furanic compounds	3-furaldehyde	C_5_H_4_O_2_	831	832	–	1.304 ± 0.141	4.526 ± 0.237	6.123 ± 0.412	11.569 ± 0.572	25.121 ± 1.460
	1-(2-furanyl)-ethanone	C_6_H_6_O_2_	911	911	–	0.039 ± 0.003	0.083 ± 0.007	0.134 ± 0.015	0.212 ± 0.019	0.734 ± 0.065
	5-methyl-2-furancarboxaldehyde	C_6_H_6_O_2_	960	965	–	0.037 ± 0.005	0.163 ± 0.013	0.476 ± 0.052	0.866 ± 0.056	3.998 ± 0.312
	2-acetyl-5-methylfuran	C_7_H_8_O_2_	1,039	1,039	–	0.007 ± 0.001	0.008 ± 0.001	0.011 ± 0.002	0.014 ± 0.002	0.028 ± 0.003
	2,5-diformylfuran	C_6_H_4_O_3_	1,079	1,076	–	0.024 ± 0.003	0.124 ± 0.014	0.241 ± 0.032	0.334 ± 0.041	0.349 ± 0.044
	1-(2-furanyl)-2-hydroxy-ethanone	C_6_H_6_O_3_	1,081	1,087	–	0.086 ± 0.009	0.582 ± 0.067	0.916 ± 0.064	1.104 ± 0.130	1.599 ± 0.018
	2-methyl-benzofuran	C_9_H_8_O	1,103	1,109	–	–	–	–	–	0.013 ± 0.002
	2-furfuryl-5-methylfuran	C_10_H_10_O_2_	1,183	1,190	–	–	–	–	–	0.004 ± 0.0006
	5-hydroxymethylfurfural	C_6_H_6_O_3_	1,229	1,233	–	0.091 ± 0.007	3.039 ± 0.291	9.246 ± 0.451	12.335 ± 0.774	13.511 ± 0.824
Pyranic compounds	2,3-dihydro-3,5-dihydroxy-6-methyl-4(H)-pyran-4-one	C_6_H_8_O_4_	1,141	1,151	–	–	0.046 ± 0.005	0.124 ± 0.015	0.118 ± 0.023	0.181 ± 0.023
	Maltol	C_6_H_6_O_3_	1,109	1,110	–	–	–	0.105 ± 0.009	0.081 ± 0.007	0.062 ± 0.008
Pyrrole	1H-pyrrole-2-carboxaldehyde	C_5_H_5_NO	1,011	1,015	–	–	–	0.047 ± 0.005	0.100 ± 0.009	0.115 ± 0.014
	2-acetyl pyrrole	C_6_H_7_NO	1,063	1,064	–	–	–	0.032 ± 0.003	0.056 ± 0.007	0.175 ± 0.015
Pyridine	3-butyl-pridine	C_9_H_13_N	1,104	1,101	–	–	–	–	–	0.012 ± 0.002

*–: Not detected*.

*^a^Kovats index relative n-alkanes (C_8_ to C_20_) on a HP-5 capillary column*.

*^b^Kovats index relative reported in the NIST database*.

### Antioxidant Activities

#### Hydroxyl Radical Scavenging Activity

**Figure 5:**
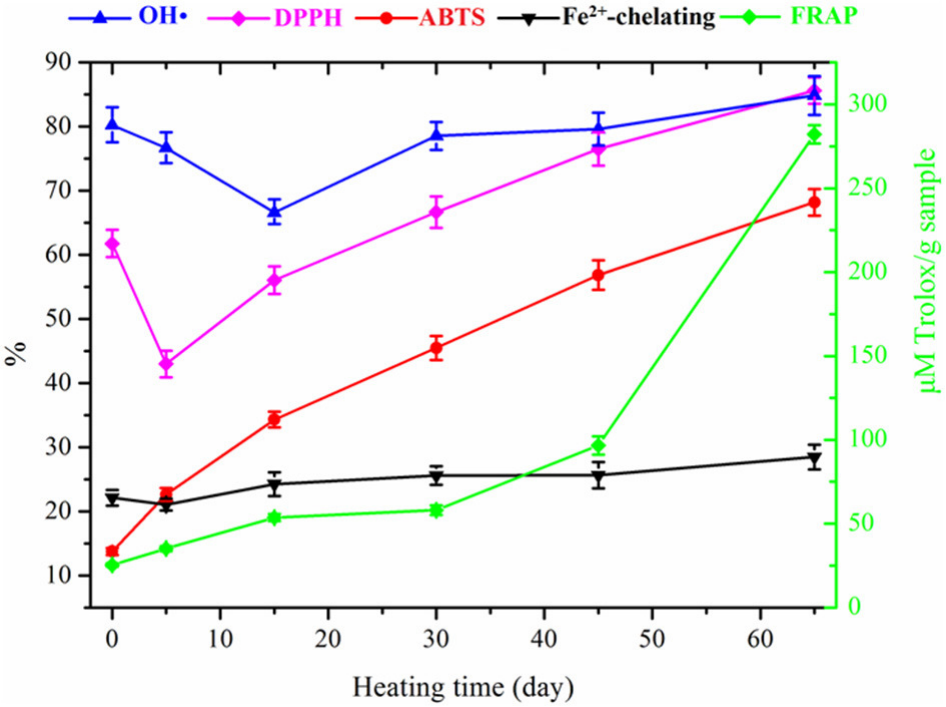
Changes in antioxidant activities of apple during 65-day HTFP process.

The scavenging activities of apple at various fermentation times against the hydroxyl radicals are shown in Figure 5. The fresh apple showed high hydroxyl radical scavenging activity of 80.2 ± 2.8%, and then, the scavenging activity of apple decreased to 60.6 ± 2.1% after 15 days' fermentation. After that, the scavenging activity of processed apple increased continuously. The scavenging rate of final black apple was 84.8 ± 3.0%. Results suggested that the hydroxyl radical scavenging activity of the black apple increased slightly compared with that of the fresh apple. The results in this study were different from those of black garlic reported by Zhang et al. (9). As reported, the black garlic showed a significantly higher scavenging activity of hydroxyl radicals than that of raw garlic.

#### DPPH Radical Scavenging Activity

The scavenging rate of DPPH radicals decreased sharply from 61.8 ± 2.1% to 43.0 ± 2.0% during the initial 5 days, increased continuously in the following fermentation stage, and then came to a maximum of 85.6 ± 2.0% with the final products. The variation trend of DPPH radical scavenging ability with fermentation time was similar as that of TPC, suggesting that a correlation existed between the two due to the demonstrated antioxidant power of polyphenols.

#### ABTS Radical Scavenging Activity

The fresh apple showed low ABTS scavenging activity of 13.7 ± 0.5%, and then the scavenging ability of processed apple increased continually to 68.2 ± 2.2% as fermentation time increased from 0 to 65 days. The ABTS radical scavenging rate of the black apple was 5-fold higher than that of the fresh apple. This result was in agreement with those of black garlic obtained by Angeles et al. (30). As reported, a higher increase of 6.5- to 9.5-folds in the antioxidant capacity of black garlic was observed.

#### FRAP Ability

As shown in Figure 5, the FRAP values of processed apple increased slowly in the initial 45 days of fermentation and then increased sharply in the last 20 days. The FRAP value of the fresh apple was 25.4 μM Trolox/g sample (DM), whereas the reducing power of the final black apple was 282.1 μM Trolox/g sample (DM). This result indicated that the reducing power of apple was significantly enhanced during the thermal processing, as the FRAP value of black apple was approximately 10-fold higher than that of fresh apple. This increase mainly occurred in the later phase of the nonenzymatic browning reaction as reported (31).

#### Fe^2+^-Chelating Ability

Results showed that the Fe^2+^-chelating ability of apple increased slowly with the increased heating time. The Fe^2+^-chelating rates of fresh apple and black apple were 22.1 ± 1.2% and 28.5 ± 1.9%, respectively. Compared with the Fe^2+^-chelating activity of fresh apple, the slight increase in the Fe^2+^-chelating activity of black apple was probably ascribed to the high MW products of nonenzymatic browning reactions such as melanoidins, which were shown to have the ability of chelating for Fe^2+^ (35).

In general, the antioxidant activities of black apple were increased compared with those of fresh apple. The increase in the antioxidant activities was statistically correlated with the TPC, physicochemical properties (K420 and ΔE), and products of MR (Table 2). Therefore, the representative antioxidant compounds in black apple could be the polyphenols. Furthermore, in the thermal process, a number of furanic compounds with high antioxidant power such as 5-hydroxymethylfurfural were generated through nonenzymatic browning reactions (36, 37). Antioxidant activities are closely related to the product's components and their structure; thus, elucidating correlative antioxidant activities with structural characteristics is of great significance (38).

**Table 2:** Correlation analysis between physiochemical characteristics and antioxidant activities.

**Correlation coefficients**	**TPC**	**K420**	**ΔE**	**Furanic compounds**	**Pyranic compounds**	**Pyrrole compounds**
ABTS	0.589*	0.891**	0.975	0.966*	0.921	0.920**
DPPH	0.866**	0.839**	0.733*	0.894**	0.836**	0.900**
•OH	0.758**	0.603**	0.383*	0.547**	0.498	0.674**
FRAP	0.802*	0.980*	0.737	0.933*	0.673	0.943*
Fe^2+^-chelating	0.660**	0.866**	0.854	0.950	0.908	0.896**

The marks * and ** indicate statistically significant difference (p < 0.05) and statistically extremely significant difference (p < 0.01), respectively.

## Conclusion

The high-temperature fermentation processing of apple caused significant changes in the physicochemical and phytochemical properties of apple, including the color, browning intensity, pH, moisture content, sugars, organic acids, polyphenolic, and volatile profiles. The antioxidant activities of black apple based on ABTS, DPPH, hydroxyl radical scavenging activity, FRAP and Fe^2+^-chelating ability were higher than those of fresh apple. The increased antioxidant activities of black apple could be ascribed to the polyphenols and products of MR. In summary, HTFP can be considered as a promising method of apple processing. It is valuable to carry out the identification of other biologically active compounds in black apple in the future study.

## Data Availability Statement

The original contributions presented in the study are included in the article/supplementary material, further inquiries can be directed to the corresponding authors.

## Author Contributions

ZZ involved in data curation, writing the original draft, writing the review, and editing. YZ involved in formal analysis, investigation, and methodology. WW involved in conceptualization, methodology, and supervision. SS involved in formal analysis. JW involved in data curation and validation. XL and FD involved in writing-review and editing. YJ involved in data curation and formal analysis. All authors contributed to the article and approved the submitted version.

## Conflict of Interest

The authors declare that the research was conducted in the absence of any commercial or financial relationships that could be construed as a potential conflict of interest.

## Publisher's Note

All claims expressed in this article are solely those of the authors and do not necessarily represent those of their affiliated organizations, or those of the publisher, the editors and the reviewers. Any product that may be evaluated in this article, or claim that may be made by its manufacturer, is not guaranteed or endorsed by the publisher.
